# Is Phosphate Solubilization the Forgotten Child of Plant Growth-Promoting Rhizobacteria?

**DOI:** 10.3389/fmicb.2018.02054

**Published:** 2018-09-03

**Authors:** Camille E. Granada, Luciane M. P. Passaglia, Eduardo M. de Souza, Raul A. Sperotto

**Affiliations:** ^1^Graduate Program in Biotechnology, University of Taquari Valley - Univates, Lajeado, Brazil; ^2^Department of Genetics, Institute of Biosciences, Federal University of Rio Grande do Sul (UFRGS), Porto Alegre, Brazil

**Keywords:** P-fertilizers, inoculation, P-solubilization, rhizobacteria, sustainable agriculture

## Chemical fertilizers × plant growth-promoting rhizobacteria

Plant growth-promoting rhizobacteria (PGPR) is a well-known group of microorganisms able to promote plant growth through enhanced biological nitrogen fixation (BNF), synthesis of plant hormones, soil nutrient solubilization (as phosphorus [P] and potassium [K]; Gupta et al., 2015), besides preventing deleterious effects of soil-borne phytopathogens (Compant et al., 2005). Due to the high importance of nitrogen (N) for plant development and the low persistence time that synthetic N fertilizer presents in the soil (Galloway et al., 2003), most of the studies are focused on microorganisms able to biologically fix atmospheric N. BNF is performed by symbiotic PGPR, which are restricted to association of leguminous plants and rhizobial isolates (e.g., *Rhizobium* spp., *Bradyrhizobium* spp., *Mesorhizobium* spp., and *Allorhizobium* spp.), or by free-living bacterial isolates (e.g., *Azospirillum* spp., *Pseudomonas* spp., *Burkholderia* spp., *Gluconacetobacter* spp., and *Herbaspirillum* spp.; Remigi et al., 2016). However, the research focused only in BNF neglects the high biotechnological potential of PGPR to agriculture.

Overuse of synthetic fertilizers and agrochemical pesticides has sustained the high crop yield and, consequently, the population growth in the last century (Stewart et al., 2005). However, environment does not sustain these practices any more. The consequences are already observed as high eutrophication of rivers, groundwater contamination, atmospheric pollution, and losses of soil quality (Stewart et al., 2005; Mondal et al., 2017). These scenarios have stimulated several agricultural researches. Replacement of synthetic N inputs by PGPR inoculation has been possible only due to the deep knowledge about BNF. It is interesting to farmers, since it reduces production costs besides being an environmental-friendly technique. However, PGPR inoculation can go further, since it presents a potential to reduce the amount of the most important synthetic inputs applied on crops, which is of paramount importance regarding fertilizers obtained from finite sources.

## Soil phosphorus (P) and P-fertilization

Phosphorus (P) is a good example of an essential nutrient for plant development derived from finite resources. P fertilizer is extracted from P-rich rock in the form of phosphate. Morocco, China, South Africa and the U.S. account for approximately 83% of the world's reserves of exploitable phosphate rock (Vaccari, 2009). Therefore, P deficiency is one of the major limitations to crop production and it is estimated that 5.7 billion hectares of land worldwide are deficient in P (Mouazen and Kuang, 2016). These numbers highlight the high importance of P fertilizers for achieving optimal crop production. Bouwman et al. (2013) estimated that annual P consumption in agriculture will increase around 2.5% per year. Considering the finite sources of P, this data and other studies indicated that a global P crisis is near (Abelson, 1999; Vaccari, 2009; Jones et al., 2015). However, none of these studies have considered the residual P in the soil (Sattari et al., 2012).

Some tropical agricultural soils are P-fixing, and the vast majority of P fertilizer added to them are adsorbed onto soil minerals [metal oxides (mainly iron and aluminum) and clay minerals], precipitated as P minerals (predominantly apatite-like minerals), and immobilized as organic P compounds (soil organic matter and phytate), making its residual P less available to crops (Martinez-Viveros et al., 2010; Hinsinger et al., 2011). Due to such P immobilization and environmental losses, producers need to apply twice or more P fertilizers than are actually needed for optimal yield production (Roy et al., 2016). It is estimated that 2–8 million tons of P fertilizer are applied to the soils every year, and ~1–4 million tons remain in the soil as a residual part. In a future scenario (2050), 4–14 million tons will be applied, and 2–7 million tons will remain in the soils (Roy et al., 2016). Considering that P fertilizer costs approximately US$ 400 per ton, around US$ 400 million to US$ 1.6 billion are lost with P fertilizers in crops around the world every year. It certainly means a substantial increase on the food prices for consumers.

## Is phosphorus solubilization the forgotten child of PGPR?

Recently, Roy et al. (2016) made a tricky question: is it possible that the increasing amount of immobilized P in the tropical agricultural soils eventually become available to plants and support crop productivity? In the case we keep using the same fertilization strategies used for many years, the answer is certainly no. However, we do believe that using adequate biotechnological approaches, the immobilized P could return to the plants in a soluble and available form. Screening of new PGP isolates for inoculant production aiming to optimize plant growth and BNF comprise an essential stage of *in vitro* phosphate solubilization analysis (Collavino et al., 2010; Souza et al., 2013, 2015; Walitang et al., 2017; Marag et al., 2018). These studies identified several bacterial isolates able to promote plant growth, improve rhizosphere area and solubilize different sources of immobilized P. Given the low mobility of P in soils, the enlargement of volume and geometry of the rhizosphere provided by PGPR inoculation determines the amount of P available to plants (Richardson et al., 2009). Therefore, inoculation of PGPR seems to be a reasonable tool to maximize such approach. Microorganisms increase the availability of inorganic P through the production of protons, organic acids, and ligands, which are ubiquitous among rhizosphere P-solubilizing microorganisms (Hinsinger et al., 2011), and also mobilize phytate (organic P) probably by phytase production (Jorquera et al., 2008). However, in greenhouse and/or field conditions, most of the studies do not evaluate different P-fertilization levels, phosphate solubilization in the soil and P uptake by the plants. The majority of the studies considers only plant agronomic parameters and plant N content in conditions with or without N fertilization.

## Reduction of P-fertilization through PGPR inoculation

Increasing P efficiency in crops without increasing or even decreasing P inputs requires a more efficient exploitation of soil microbial resources in agroecosystems. Some studies clearly report that plant inoculation with new PGPR can improve P uptake. Rudresh et al. (2005) showed that chickpea plants inoculated with *Rhizobium* sp. and *Bacillus* sp. present higher yield (two-fold) and higher P content (four-fold) in the grain. Vyas and Gulati (2009) and Granada et al. (2013) demonstrated that inoculation of maize (*Zea mays*) with *Pseudomonas* spp., and *Lupinus albescens* plants with free-living *Sphingomonas* sp. results in almost three-fold increases in their shoot P contents, respectively. Studying wheat (*Triticum aestivum* L.) plants, Kumar et al. (2014) showed that inoculation of *Bacillus megaterium, Arthrobacter chlorophenolicus*, and *Enterobacter* improves grain yield and the amount of P in the straw and grain up to two-fold in greenhouse and field experiments. Thus, it is already known that inoculation of efficient P-solubilizer bacteria significantly improve P absorption by plants, even though most of the experiments use the recommended P fertilizer dose, and reduction of the P-fertilization has not been evaluated.

Khalafallah et al. (1982) developed an important work inoculating *Vicia faba* plants with P-solubilizing bacteria. This work showed the possibility of reducing the P-fertilization up to 50%, once plants that received half of the recommended P-fertilizer dose presented similar plant dry weight and P-uptake when compared to plants that received usual P-fertilizer dose. More recently, Lavakush et al. (2014) observed the same potential in rice plants inoculated with the P-solubilizing bacteria *Azotobacter chroococcum, Azospirillum brasilense*, and combined *Pseudomonas* spp. culture. Inoculated rice plants presented similar performance in plant height, panicle length, grain number per panicle and grain yield when fertilized with 30 and 60 kg P ha^−1^ in a greenhouse experiment. Dutta and Bandyopadhyay (2009) showed that reduction of up to one-third in P-fertilization of chickpea plants (inoculated with P-solubilizing *Pseudomonas* sp.) did not cause any decrease in plant development parameters.

**Figure 1:**
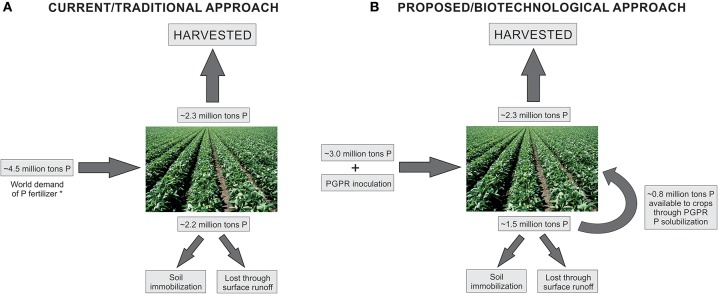
Schematic model of current/traditional approach **(A)** and proposed/biotechnological approach **(B)**. *On the current agricultural approach, world demand of P fertilizer is approximately 4.5 million tons, according to FAO (http://www.fao.org/3/a-i6895e.pdf). From these, 2.2 million tons are unavailable to crops (soil immobilization or surface runoff), and 2.3 million tons are harvested with the crops. On the proposed biotechnological approach, we suggest the reduction of up to 33% on the P fertilizer dose applied on the soil, along with PGPR inoculation. Such reduction on P fertilizer together with PGPR inoculation would result in less P unavailable to the crops. Nearly half of such unavailable P can be further solubilized by PGPR and uptaked by the crops, resulting in the same 2.3 million tons of harvested P (adapted from Roy et al., 2016).

Therefore, PGPR inoculation can probably be used to reduce P-fertilization, being an excellent biotechnological tool. However, this research area is neglected by researches and certainly needs more investigation. All plant species are able to establish a relationship with some PGPR, and the selection of new bacterial isolates, able to solubilize different forms of P *in vitro*, is an important and necessary first step. We hope the results obtained in greenhouse and field inoculation experiments with selected P-solubilizing bacterial isolates and plant species subjected to reduced amounts of P-fertilizer could serve as an alert to producers about the high costs of normally used fertilization strategies, the concerns about finite P sources, and the environmentally friend biotechnological option of using PGPR. Based on previous works which addressed P-solubilization potential by PGPR inoculation in plants (mainly Khalafallah et al., 1982; Dutta and Bandyopadhyay, 2009; Kumar et al., 2014; Lavakush et al., 2014; Anzuay et al., 2015; Kaur and Reddy, 2015), we consider that an average reduction of 33% in P-fertilization could be achieved with the use of high efficient P-solubilizing bacterial isolates as crop inoculants, as indicated on the proposed biotechnological approach in Figure 1. Therefore, future experiments need to be specifically designed for such purposes. Considering the complexity of these mechanisms, an interdisciplinary approach taking into account molecular, biochemical, physiological, and agronomic parameters has a good probability to generate positive results. We have a long way to cross until reaching similar knowledge and applicability achieved by bacterial inoculants regarding the reduction of N-fertilizers. However, reasonable use of environmental resources should be the basis for modern and sustainable agriculture development.

## Author contributions

All authors listed have made a substantial, direct and intellectual contribution to the work, and approved it for publication.

### Conflict of interest statement

The authors declare that the research was conducted in the absence of any commercial or financial relationships that could be construed as a potential conflict of interest.
